# Supplementary figures and images for: Identification of fresh leaves of Anji White Tea: S-YOLOv10-ASI algorithm fusing asymptotic feature pyra-mid network
Source: PLoS One. 2025 Jul 2;20(7):e0325527. doi: 10.1371/journal.pone.0325527 (PMC12221049; doi:10.1371/journal.pone.0325527)

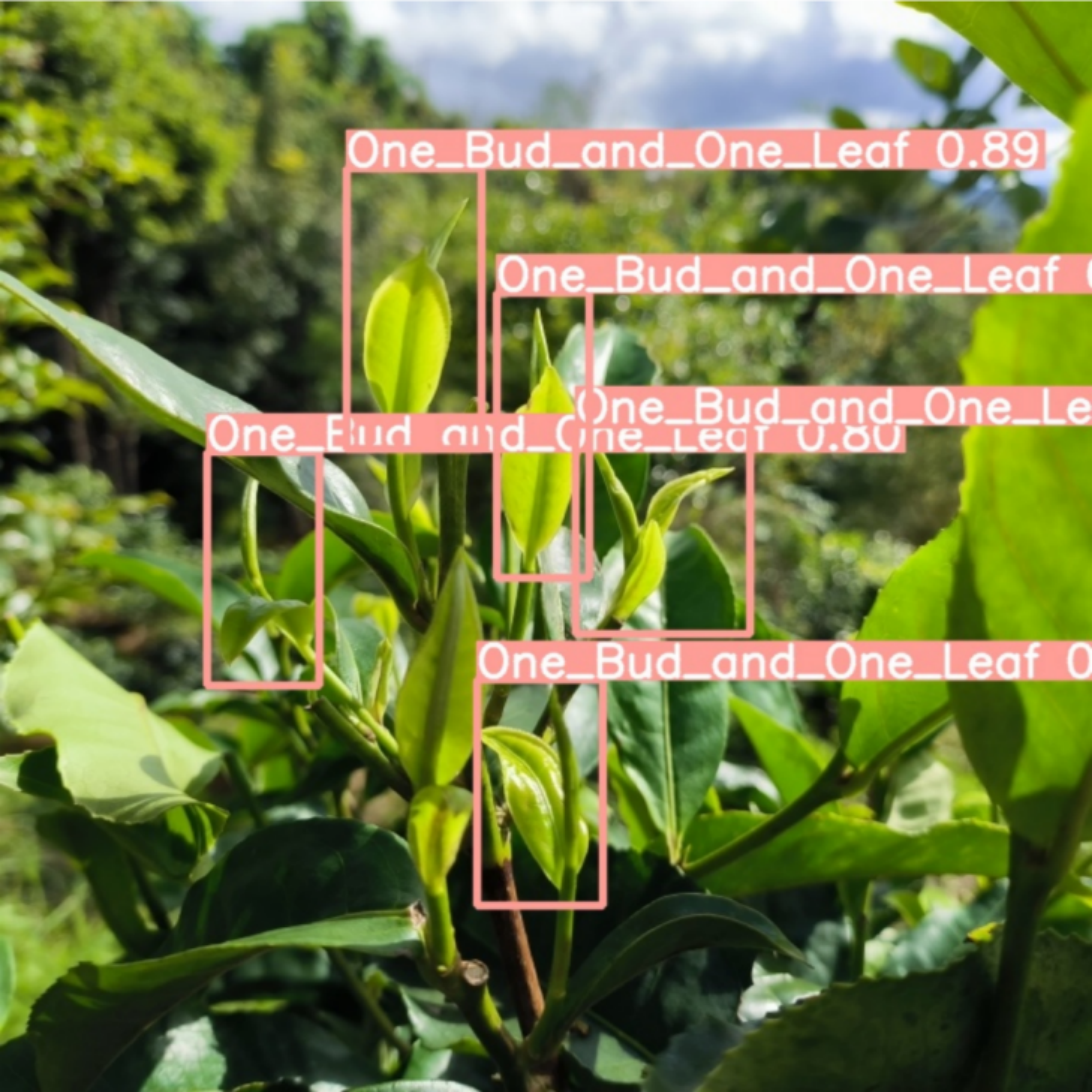

Supplement: S1 File — (TIFF) [file pone.0325527.s001.tiff]
