# Supplementary material for: Identification of fresh leaves of Anji White Tea: S-YOLOv10-ASI algorithm fusing asymptotic feature pyra-mid network
Source: PLoS One. 2025 Jul 2;20(7):e0325527. doi: 10.1371/journal.pone.0325527 (PMC12221049; doi:10.1371/journal.pone.0325527)
Supplement: Attachment — Submitted filename: Response_to_Reviewers_auresp_1.docx [file pone.0325527.s002.docx]

Thanks very much for your time to review this manuscript. I really appreciateyou’re your comments and suggestions. We have considered these comments carefully and triedour best to address every one of them. And the corresponding part in the text has been modified using red font.

**Review 1:**

1. **Clarity in Methodology: The paper introduces a novel S-YOLOv10-SIC algorithm for identifying tea leaves, which improves upon YOLOv10 through several advanced techniques. However, the detailed steps in the methodology need clearer descriptions. Some parts, like “the fusion of non-adjacent layers” and “the integration of the slice-assisted super-reasoning algorithm”, are mentioned but not fully explained for replication.**

**Modification instructions:** We have been changed in accordance with the advice given. “In terms of the improvement to Space-to-Depth Convolution, the focus is mainly on the layers following the second to fifth convolutional layers in the backbone. For the improvement of the Asymptotic Feature Pyramid Network, the emphasis is primarily on the neck part. The Slicing Aided Hyper Inference Algorithm is located between the input and the backbone during the model detection stage.”

2. **Experimentation: The explanation of the dataset and augmentation process could be elaborated upon. For instance, the reason behind choosing lighting conditions and scaling parameters is unclear.**

**Modification instructions:** The operation is primarily implemented through Python programming, and the reason for selecting the random range has been supplemented in the main text. “The random range is mainly set based on the actual environmental variations in the tea garden.”

3. **Technical Contributions: The Inner-IoU optimization and the Asymptotic Feature Pyramid Network are innovative, but the equations and their implementation are not described in a user-friendly manner. The equation-heavy presentation can be daunting for readers unfamiliar with these concepts.**

**Modification instructions:** Thank you for your valuable feedback on the Asymptotic Feature Pyramid Network and Inner-IoU sections of our paper. We have noted your suggestions for improving the mathematical formulations of Inner-IoU optimization and AFPN, especially regarding the readability of the formulas and their implementation friendliness. In response, we have made several adjustments, combining images with formulas to present these concepts in a more accessible way, making the technical content easier to understand for a wider audience. For example, “Inner-IoU primarily evaluates the overlap between the predicted box and the ground truth box by calculating the ratio of the green region to the yellow region.”

4. **Figures and Visual Aids: The diagrams and figures provided are generally helpful, but a few, such as Figure 3 (improved YOLOv10 network) and Figure 7 (Slicing Aided Hyper Inference Algorithm), lack sufficient labeling to make them easily interpretable on their own.**

**Modification instructions:** Thank you for your valuable feedback regarding the diagrams and figures in the paper. In Figure 3, we differentiated the various modules using different colors to help readers better understand the structure. In Figure 7, we have added supplementary information: “During the slicing phase, the data is divided into smaller chunks to reduce the computation and memory usage of a single inference. In the hyper-inference phase, the algorithm performs inference operations on each slice in parallel, which speeds up the overall process. Finally, in the result fusion phase, the inference results from each slice are combined to ensure the accuracy of the output.”

5. **Writing and Formatting: Some sections of the manuscript, especially in the discussion and conclusion, repeat the same information. This detracts from the flow and depth of insight. For example, the improvements in mAP, precision, and recall are mentioned multiple times without adding new insights in each instance.**

**Modification instructions:** Have been changed in accordance with the advice given. We have removed the repetitive parts.

**Review 2:**

6. **The paper requires English language editing, as the current expression is unclear.**

**Modification instructions:** Thank you for your feedback on the language quality of the paper. I acknowledge that some parts of the text may lack clarity, and I will make sure to undergo thorough English language editing to improve expression and readability. I will revise sentences and sections that are unclear to ensure better coherence and precision. Your comments are greatly appreciated, and I will address this issue promptly to enhance the overall quality of the paper.

7. **Thank you for your valuable comments on the Introduction section. I appreciate your observation regarding the lack of clarity in explaining the relationship between the picking robot and the detection algorithm. I will revise the Introduction to better establish this connection, making it clear how the detection algorithm is integral to the robot's operation. This clarification will be added between the first and second paragraphs, ensuring that readers can easily understand the context.**

**Modification instructions:** Thank you for your valuable comments on the Introduction section. I appreciate your observation regarding the lack of clarity in explaining the relationship between the picking robot and the detection algorithm. I will revise the Introduction to better establish this connection, making it clear how the detection algorithm is integral to the robot's operation. This clarification will be added between the first and second paragraphs, ensuring that readers can easily understand the context. “With the rapid development of deep learning algorithms in recent years, the application of mechanized and intelligent production technologies in the agricultural field has achieved remarkable success.”

8. **The abstract and introduction repeatedly mention improving convergence speed, but no specific time data is provided in the text. Similarly, the claim of reducing image information loss lacks quantitative support.**

**Modification instructions:** Thank you very much for your suggestion. We have made the addition in the Model Analysis section. Compared to YOLOv10, the improved YOLOv10-ASI achieves earlier convergence. We have also made corresponding revisions to the description of the loss of image information. “As shown in Fig 9, the three types of loss functions of the YOLOv10-ASI network decrease faster than the YOLOv10 network, and the loss function of the YOLOv10-ASI network is significantly slowed down around 500 rounds, which is about 200 rounds earlier than the original YOLOv10 network. After 800 training rounds, the loss function of the YOLOv10-ASI network stabilized, achieving this milestone approximately 100 rounds earlier than the original YOLOv10 network.” “Classification Loss and Distribution Focal Loss showed minimal variation in the training set compared to the original YOLOv10, while Bounding Box Regression Loss declined by over 30%. In the validation set, Classification Loss and Bounding Box Regression Loss each decreased by more than 60%, and Distribution Focal Loss experienced a reduction of around 10%.”

9. **Does the proposed improved YOLOv10 compare with related research, such as the same team’s improvement of YOLOv5?**

**Modification instructions:** Thank you for your thoughtful comment.We have added in the original text the improvements brought by this algorithm modification compared to our original YOLOv5 improvement. "Compared to the 94.52% mAP value of our team's previous ShuffleNetv2-YOLOv5-Lite-E network, it shows an improvement of 2.4%."

10. **The colors in the modules of Figure 3 are confusing for the reviewer. The same modules should be shown with the same colors. Additionally, the "Neek" part is unclear, and the feature flow out seems to be misdirected or piled up.**

**Modification instructions:** Thank you for your valuable feedback regarding the diagrams and figures in the paper. In Figure 3, we differentiated the various modules using different colors to help readers better understand the structure.

11. **Figure 5 does not match Figure 3; Figure 3 shows 3 outputs from the backbone, while Figure 5 shows 4.**

**Modification instructions:** Have been changed in accordance with the advice given. This is because, in the original technology, the feature progressive pyramid structure was designed with 4 outputs. In order to integrate it into the YOLO algorithm, we have modified it to 3 outputs, and the corresponding images have been updated accordingly.

12. **The ASF-1 module in Figure 5 is not reflected in Figure 3.**

**Modification instructions:** Have been changed in accordance with the advice given. This is because we did not use this part; it was included merely for the convenience of readers to reference the original paper on the feature progressive pyramid structure. We have now removed the unused portion.

13. **There are two Figure 3s in the paper; one should be Figure 6.**

**Modification instructions:** Have been changed in accordance with the advice given.

14. **In Figure 9, the loss function could be zoomed in locally for better analysis. In the second validation set of YOLOv10, the loss reaches 40, which is an outlier compared to the other data, but there is no corresponding analysis of this anomaly.**

**Modification instructions:** Have been changed in accordance with the advice given. The reason the loss value reached 40 in the first round is that we trained without using pre-trained weights. However, it is clear that in the second and third rounds, the loss function started to decrease linearly.

**Review 3:**

15. **The language and format are terrible and insufferable. The authors must check the manuscript carefully to remove the typos, improve the language and format.**

**Modification instructions:** Thank you for your valuable feedback. We sincerely apologize for the issues with language and formatting in the manuscript. We have carefully reviewed the paper and made revisions to improve the clarity and flow of the language. Typos have been corrected, and the formatting has been aligned with the required standards to ensure a more professional presentation. We appreciate your patience and understanding, and we hope the revised manuscript meets your expectations.

16. **The Abstract is too long and confused.**

**Modification instructions:** Thank you for your insightful feedback. We understand that the Abstract needs to be concise and clear. We have revised it to remove redundant information and ensure it accurately summarizes the key points of the paper in a more succinct and coherent manner. We hope the revised Abstract aligns better with the expectations for clarity and brevity.

17. **Some figures are unclear. The details of the blocks, such as the parameters, should be clearly defined and explained directly in the figures even they have been explained in text. Please provide the information as much as possible, then the readers can get the details from each separate figure easily even without the text.**

**Modification instructions:** Thank you for your constructive feedback. We appreciate your point about the clarity of the figures. We have revised the figures to ensure that the details, such as parameters and other relevant information, are clearly defined and explained directly within the figures. This will allow readers to understand the details of each figure without needing to refer to the text. We have also added additional annotations and labels where necessary to improve clarity and accessibility.

18. **Please use bold font to label the best results in all tables.**

**Modification instructions:** Thank you for your helpful suggestion. We have reviewed all tables in the manuscript and have applied bold font to highlight the best results, as per your recommendation. This will help the readers easily identify the key outcomes. We appreciate your feedback and believe that this change enhances the overall readability and presentation of the results.

19. **Please provide and label the reference indices of the compared methods in the figures and tables, and then the readers can judge whether the compared methods are SOTA.**

**Modification instructions:** Thank you for your insightful suggestion. We agree that providing and labeling the reference indices of the compared methods in the figures and tables will help readers easily evaluate whether the methods are state-of-the-art (SOTA). We have now updated the relevant figures and tables to include the reference indices for all the compared methods. This will enable readers to assess the performance of each method in the context of existing research.
