# Supplementary material for: Identification of fresh leaves of Anji White Tea: S-YOLOv10-ASI algorithm fusing asymptotic feature pyra-mid network
Source: PLoS One. 2025 Jul 2;20(7):e0325527. doi: 10.1371/journal.pone.0325527 (PMC12221049; doi:10.1371/journal.pone.0325527)
Supplement: Attachment — Submitted filename: Response_to_Reviewers_auresp_3.docx [file pone.0325527.s004.docx]

Thanks very much for your time to review this manuscript. I really appreciate your comments and suggestions. We have considered these comments carefully and tried our best to address every one of them. And the corresponding part in the text has been modified using red font.

**Additional Editor Comments:**

1. **This article has been revised and improved. However, there may be some repeated expressions in some sentences. Please proofread and revise them carefully.**

**Modification instructions:** Thank you for your valuable review comments and suggestions! We have made detailed revisions to the manuscript and carefully reviewed all sentences for any possible redundant expressions. All revisions are marked in the 'Revised Manuscript with Track Changes' section, with red font used for differentiation to facilitate your review. Once again, thank you for your help, and we look forward to your further feedback!

**Reviewer 1:**

2. **The model stil is not properly and clearly described and is not understandable to readers, especially those who are not familiar with the methods and algorithms. The authors' explanations to the reviewers, although commendable, should be understood by the readers.**

**Modification instructions:** Thank you for the valuable comments from you and the reviewers. We have made detailed revisions addressing the issue of redundancy in the manuscript as raised by the reviewers, ensuring that there are no unnecessary repetitions in the sentences and content. In addition, we have further clarified and improved the description of the model to ensure that even readers who are not familiar with the relevant methods and algorithms can easily understand. During the revision process, we have simplified the expression as much as possible to make it more comprehensible. All revisions are marked in the 'Revised Manuscript with Track Changes' section, with red font used to facilitate your and the reviewers' review.
